# The Impact of Visualization Dashboards on Quality of Care and Clinician Satisfaction: Integrative Literature Review

**DOI:** 10.2196/humanfactors.9328

**Published:** 2018-05-31

**Authors:** Saif Sherif Khairat, Aniesha Dukkipati, Heather Alico Lauria, Thomas Bice, Debbie Travers, Shannon S Carson

**Affiliations:** ^1^ University of North Carolina at Chapel Hill Chapel Hill, NC United States

**Keywords:** intensive care unit, visualization, Dashboard, cognitive load, information overload, usability, user interface design, health information technology, electronic health record

## Abstract

**Background:**

Intensive Care Units (ICUs) in the United States admit more than 5.7 million people each year. The ICU level of care helps people with life-threatening illness or injuries and involves close, constant attention by a team of specially-trained health care providers. Delay between condition onset and implementation of necessary interventions can dramatically impact the prognosis of patients with life-threatening diagnoses. Evidence supports a connection between information overload and medical errors. A tool that improves display and retrieval of key clinical information has great potential to benefit patient outcomes. The purpose of this review is to synthesize research on the use of visualization dashboards in health care.

**Objective:**

The purpose of conducting this literature review is to synthesize previous research on the use of dashboards visualizing electronic health record information for health care providers. A review of the existing literature on this subject can be used to identify gaps in prior research and to inform further research efforts on this topic. Ultimately, this evidence can be used to guide the development, testing, and implementation of a new solution to optimize the visualization of clinical information, reduce clinician cognitive overload, and improve patient outcomes.

**Methods:**

Articles were included if they addressed the development, testing, implementation, or use of a visualization dashboard solution in a health care setting. An initial search was conducted of literature on dashboards only in the intensive care unit setting, but there were not many articles found that met the inclusion criteria. A secondary follow-up search was conducted to broaden the results to any health care setting. The initial and follow-up searches returned a total of 17 articles that were analyzed for this literature review.

**Results:**

Visualization dashboard solutions decrease time spent on data gathering, difficulty of data gathering process, cognitive load, time to task completion, errors, and improve situation awareness, compliance with evidence-based safety guidelines, usability, and navigation.

**Conclusions:**

Researchers can build on the findings, strengths, and limitations of the work identified in this literature review to bolster development, testing, and implementation of novel visualization dashboard solutions. Due to the relatively few studies conducted in this area, there is plenty of room for researchers to test their solutions and add significantly to the field of knowledge on this subject.

## Introduction

### State of the Problem—Critical Patient Population

Intensive Care Units (ICUs) in the United States admit more than 5.7 million people each year [1]. The ICU level of care helps people with life-threatening illness or injuries and involves close, constant attention by a team of specially-trained health care providers [2]. ICU patients require frequent assessment and have a greater need for technological and clinical support compared to non-ICU patients [1]. Important metrics in the ICU range from simple vital sign monitoring and laboratory data to mechanical ventilator support, vasoactive medications, and even complete circulatory support, depending on the unique needs of specific patients. Although ICU patients receive care for a wide variety of disease states, the leading causes of death in the ICU are multi-organ system failure, cardiovascular failure, and sepsis [2]. Delay between condition onset and implementation of necessary interventions can dramatically impact the prognosis of a patient with one of these life-threatening diagnoses.

### Electronic Health Record Usability

EHR use has increased nationwide; however, the question remains whether EHRs are being used in an effective and efficient way that improves clinical workflow and health outcomes [3,4]. A systematic review and meta-analysis intended to evaluate effects of health information technology in the hospital and ICU on mortality, length of stay, and cost found significant interstudy and intrastudy variability. The study demonstrated that more research is needed with standardized interventions and endpoints to evaluate EHR use and implementation. Currently, no conclusion can be made regarding the effect of health information technology on inpatient and ICU outcomes such as mortality, length of stay, and cost [4].

### Information Overload

In 2013, Singh, Spitzmueller, Petersen, Sawhney, and Sittig conducted a cross-sectional study of primary care providers to evaluate predictors of missed test results in the setting of electronic health record (EHR) alerts. Of the nearly 2,600 respondents, 87% perceived the quantity of alerts they received to be excessive, 70% reported receiving more alerts than they could effectively manage, 56% reported that the current EHR notification system made it possible for practitioners to miss test results, and 30% reported having personally missed test results that led to care delays [5]. To address the high volume of metrics used and the time-sensitive nature of responding to changes in a critically ill patient's condition, a tool that improves ICU display and retrieval of key clinical information has great potential to benefit patient outcomes.

### Proposed Solution

Visualization is a field of study concerned with the transformation of data to visual representations, where the goal is the effective and efficient cognitive processing of data [6]. Use of visualization techniques in the clinical setting have the potential to improve data display and cognitive processing of data, reducing cognitive overload among clinicians [6]. Information visualization involves the transformation from lower-level data to visual representations of meanings extracted from the data [6]. Extraction is by either a computational process or a human transcription process, the aim of which is to explore data and create new insights [6].

Some guidelines for the development of an information visualization solution include:

- Apply realistic techniques to enhance mapping of data elements to visual objects.
- Minimize user actions to accomplish a goal.
- Provide flexibility in the ways to achieve the same goal.
- Provide functionality to represent additional information.
- Spatially organize the visual layout.
- Consistently apply design choices.
- Place minimal cognitive load on the user.
- Provide users with information on alternatives when several actions are available.
- Remove extraneous or distracting information.
- Consider means to reduce the data set [6].

A dashboard is a data-driven clinical decision support tool capable of querying multiple databases and providing a visual representation of key performance indicators in a single report [7]. The utility of a dashboard comes from its ability to provide a concise overview of key information [7]. Applied to the intensive care unit, a dashboard allows clinicians to quickly identify changes in the patient's condition that require intervention. The clinician can choose to dive deeper into the EHR data or refer to the dashboard at a later point to review changes. Depending on the design of the dashboard, features such as alerts and documentation reminders can help clinicians improve compliance with best practice guidelines and organizational standards [7].

### Purpose of this Literature Review

The purpose of conducting this literature review is to present previous research on the use of visualization dashboards to improve efficiency, clinician satisfaction, patient safety and accuracy in the clinical setting. This evidence can be used to guide the development, testing and implementation of new solutions to optimize the visualization of clinical information.

## Methods

### Inclusion Criteria

Articles were included if they addressed the development, testing, implementation, or use of a visualization dashboard solution in a health care setting. An initial search was conducted of literature on dashboards only in the intensive care unit setting, but there were limited articles found that met the inclusion criteria. A secondary follow-up search was conducted to broaden the results to any health care setting. Ideally, the article would compare outcomes with the novel solution to outcomes prior to or without the novel solution. However, articles were not excluded simply due to lack of a specific comparison. Articles should contain quantitative or qualitative outcomes related to clinician satisfaction, cognitive overload, or patient outcomes. Initially, abstracts were scanned to identify if articles were relevant to the specified research questions.

**Figure 1:**
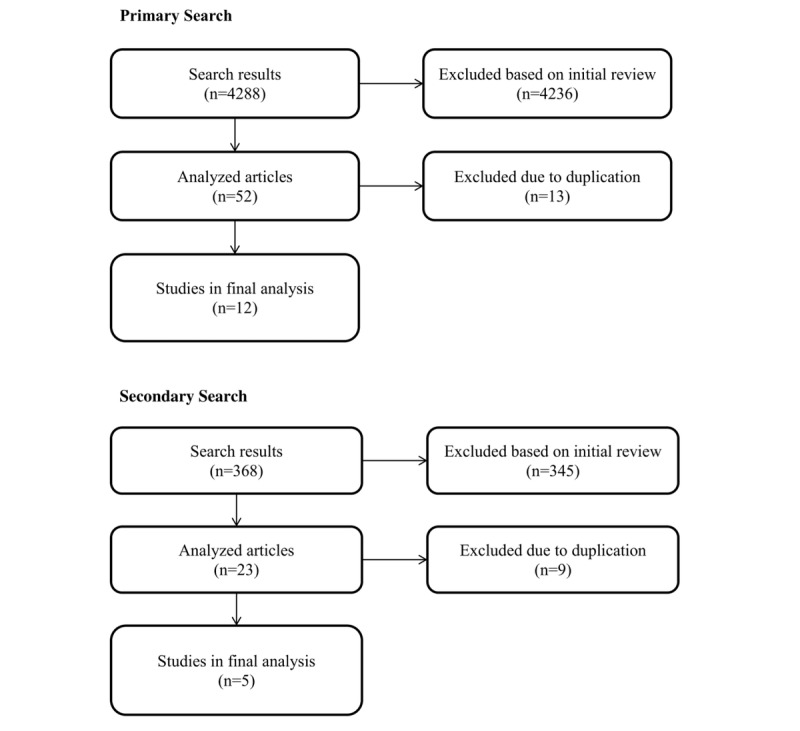
Literature review process.

### Exclusion Criteria

Due to the specificity and novel nature of this topic, no filters were applied to the query. This means that articles were not excluded solely based on type, publication date, or country of origin. However, articles were excluded if there was not an English version of the article available. Articles were excluded if review of the abstract and full text revealed the article did not address at least one of the specified research questions and meet the inclusion criteria.

### Databases

Databases selected for this search were PubMed, PMC, CINAHL, and EMBASE, all of which are health sciences journal article databases.

### Initial Search Terms—Intensive Care Unit Only

To capture alternative ways of denoting the terms of interest, the query of (“electronic medical record” OR “electronic health record” OR EMR OR EHR) AND (“visualization” OR “dashboard” OR “design” OR “interface”) AND (“intensive care” OR “ICU” OR “critical care” OR “CCU”) was used for the initial search. Abstracts were screened for relevance to the intended investigation. Articles with relevant abstracts were then read in entirety to further screen the relevance and quality of the data.

Searching the above query into PubMed returned 151 results, 14 of which were analyzed, and eight of which were relevant, quality results for the final analysis. PMC returned 3405 results, 13 of which were analyzed, five of which were excluded due to duplication, and three of which were included in the final analysis. CINAHL returned 86 results, seven of which were analyzed, two of which were excluded due to duplication, and 0 included in the final analysis. EMBASE returned 646 results, 18 of which were analyzed, six of which were excluded due to duplication, and one of which was included in the final analysis. Therefore, a total of 12 articles were obtained from this primary search towards the final analysis [8-19].

### Follow-up Search Terms—Any Health Care Setting

Because of the limited number of results obtained with the initial search, a secondary search was completed using the query of visualization AND dashboard. The purpose of the secondary search was to broaden the search to a visualization dashboard solution in any health care setting, as opposed to only the intensive care unit setting. The same databases and process of screening articles were maintained from the initial search process.

Searching the above query into PubMed returned 24 results, seven of which were analyzed, one of which was excluded due to duplication, and four of which were relevant, quality results for the final analysis. PMC returned 311 results, 10 of which were analyzed, five of which were excluded due to duplication, and one of which was included in the final analysis. CINAHL returned three results, two of which were analyzed, none of which were excluded due to duplication, and none of which were included in the final analysis. EMBASE returned 30 results, four of which were analyzed, three of which were excluded due to duplication, and none of which were included in the final analysis. Therefore, a total of five articles were obtained from the secondary search towards the final analysis, Figure 1 [20-24].

### Implications of Query Results

The initial and follow-up searches returned a total of 17 articles that were analyzed for this literature review [8-24]. The limited results reflect the novel status of this area of research. Supporting information from information and library science databases will be useful in the analysis steps as much of this work involves development, testing, and implementation of a novel software solution.

## Results

The dashboard solutions that were identified in the 17 articles are presented with organization by study findings related to efficiency, quality and safety, accuracy, and user satisfaction. Some solutions were discussed in multiple articles, whereas others were unique to a single article. Table 1 presents the sample size, metrics of interests, results and findings for each study.

### Efficiency

The Ambient Warning and Response Evaluation (AWARE) system was tested in two articles included in this literature review [8,9]. AWARE is an ICU-specific patient viewer and monitoring system that was developed at Mayo Clinic [8]. AWARE is a superstructure for existing EHR. The development of this tool was guided by clinicians and based their information needs [8]. Pickering et al (2015) used a step wedge cluster randomization trial to demonstrate a decrease in time spent on pre-round data gathering using the AWARE system [9]. Compared to the existing EHR, AWARE was reported to improve information management (data presentation format and efficiency of data access) and make the task of gathering data for rounds significantly less difficult and mentally demanding [9].

Scripps Clinic and Green Hospital used a rapid-cycle evaluation process to develop the algorithms, alert systems, and interfaces intended to facilitate patient-provider interactions and determination of treatment plans [20]. Brooke's Standardized Usability Tool was used to evaluate usability and two independent appraisers reviewed the think aloud sessions for usability themes [20]. Results pointed to positive results regarding usability and efficiency to identify pertinent components in the patient's plan of care with use of the prototype [20].

**Figure 2:**
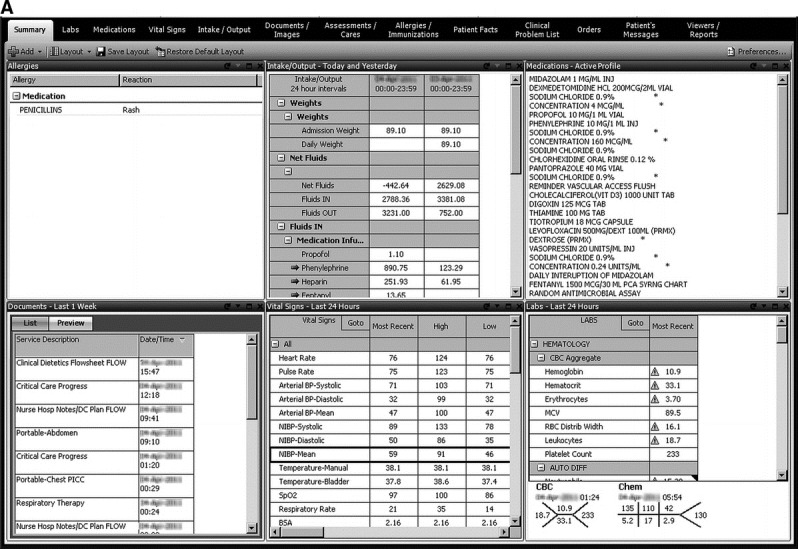
“Elements of data are pulled from across the entire electronic medical record and are organized in the systems based manner most commonly encountered in the study's intensive care unit setting.” [10].

Ahmed et al (2011) evaluated a novel .NET-based application by conducting a randomized crossover study [10]. This study demonstrated improved workload (using NASA-task load index), decreased time to task completion, and decreased number of errors of cognition. Additionally, the standard EHR contained a much larger data volume compared with the novel user interface [10]. An image of this patent-pending dashboard is shown in Figure 2.

**Figure 3:**
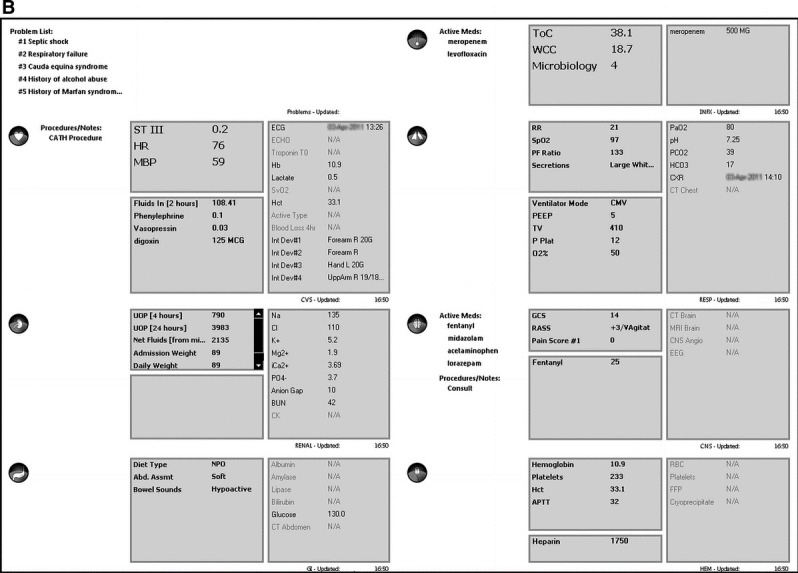
“Elements of data are pulled from across the entire electronic medical record and are organized in the systems based manner most commonly encountered in the study's intensive care unit setting.” [10].

Koch et al (2013) evaluated nurses’ situation awareness and task completion time using an integrated information display compared to traditional displays [11]. Task completion time (response time from seeing the question to submitting the answer) was measured using paper prototypes of both displays [11]. Task completion times were nearly half with integrated displays compared to traditional displays [11]. Figure 3 demonstrates a screenshot of the integration of information displays that was used by Koch et al (2013).

Farri et al (2012) carried out three iterations of planning, risk analysis, design, and evaluation of an EHR prototype. This user interface contained specific functionalities for clinical documents [12]. They used a spiral model for software development and the EHR system user interface framework of the Veterans Affairs computerized patient record system (VistA CPRS) [12].

The researchers used a mixed methods approach to evaluate a sample of eight medical interns as they synthesized EHR clinical documents in four pre-formed clinical scenarios [12]. Despite the non-significant difference in total times to task completion the researchers observed shorter times for two scenarios with the visualization tool. This may suggest that the timesaving benefits may be more evident with certain clinical processes [12].

Dolan et al (2013) used a mixed quantitative and qualitative evaluation process to evaluate their dashboard prototype [21]. The researchers observed the time participants spent using the dashboard before choosing a preferred drug, ease of use, acceptability, decisional conflict, and an open-ended qualitative analysis [21]. Qualitative findings were positive, suggesting potential for informed decision making and patient centered care [21].

**Table 1:** Study characteristics and results.

Study	Metric of Interest	Sample (n)	Result	Findings
Ahmed et al (2011) [10]	Accuracy, Efficiency	160	Errors Per Provider—Standard: 0.5, AWARE^a^: 2, *P*=.01; Workload-Standard: 38.8, AWARE: 58, *P*<.001; Time-Standard: 145, 125, 129, 112 s, AWARE: 93, 60, 68, 54, *P*<.001, data volume 1008 vs 102.	Less errors per provider, decreased time to task completion for 4 patients, improved workload (NASA-TLX^b^) scores shown after using the visualization tool.
Farri et al (2012) [12]	Accuracy, Efficiency	8	Accuracy: Missing data 2.3 (SD 1.2) with the visualization tool, 6.8 (SD 1.2) without the visualization tool, *P*=.08, accurate inferences 1.3 (SD 0.3) vs. 2.3 (SD 0.3), *P*=.09.	Lower risk missing (unretrieved) patient information with the visualization tool. More accurate inferences. Not statistically significant. Time decreased in two visualization scenarios.
Koch et al (2013) [11]	Accuracy, Efficiency	12	Time-Standard: 42.1 s, Dashboard: 26.0 s, *P*<.001; Accuracy-Standard: 1.8%, Dashboard: 85.3%, *P*<.001.	Nurses had task completion times were nearly half with integrated displays compared to traditional displays.
Clarke et al (2016) [20]	Accuracy, Efficiency, Satisfaction	Mock patients: 15	Analysis of data unavailable.	Discussion of Brooke's Standardized Usability Tool to evaluate usability themes. Examined accuracy and efficiency of Heart Team in identifying pertinent components of patient plan of care.
Faiola et al (2015) [13]	Accuracy, Efficiency, Satisfaction	12	Time-experimental group was faster in answering two questions: [Q3] t(10)=3.11, *P*=.01, r=.70; [Q4] t(10)=3.65, *P*=.004, r=.76; Accuracy-experimental (mean .65, SD .30), control groups (mean .58, SD .36), χ2(1,12)=5.04, *P*=.03.	Clinical decision-making accuracy was higher when using the visualization dashboard. Faster decision-making on 2/8 questions. Qualitative discussion of potential positive impact of MIVA^c^ 2.0
Pickering et al (2015) [9]	Efficiency	Pre: 80, Post: 63	Time on preround data gather; Pre: 12 min, Post: 9 min.	Improved efficiency of information management and data presentation; reduced mental demand.
Dolan et al (2013) [21]	Efficiency, Quality or Safety	25	Mean time interacting with the dashboard=4.6 min. No comparison group.	Interactive clinical decision dashboard are capable of fostering informed patient decision making and patient centered care.
Pageler et al (2014) [15]	Efficiency, Quality or Safety	64	Increased compliance with dressing changes from 87% to 90% (*P*=.003); cap changes 87% to 93% (*P*<.001); port needle changes 69% to 95% *P*<.001); decreased compliance with insertion bundle compliance 67% to 62% *P*=001); 2.6 CLABSIs^d^ per 1000 line-days before intervention to 0.7 CLABSIs per 1000 line-days.	Improved compliance with an evidence-based, pediatric-specific catheter care bundle.
Hagland (2010) [17]	Quality/Safety	N/A^e^	No quantifiable data.	Potential to improve patient safety, communication and clinician workflow.
Shaw et al (2015) [16]	Quality or Safety	450	Time-Pre: 393 min, Post: 202 min, *P*=.05, Quality/Safety-Decreased urinary catheter 16 to 11, *P*=.01	The median time from PICU^f^ admission to obtaining treatment consent decreased by 49%. Patients with catheter in place >96 hours decreased from 16 to 11.
Dziadzko et al (2016) [8]	Satisfaction	361	Pre: 15 min, Post: 12 min, *P*=.03.	Less time spent on gathering data using the visualization tool
Bakos et al (2012) [14]	Satisfaction, Quality or Safety	N/A	No quantifiable data.	Increased usage showed clinician satisfaction, benefits for staff per interviews, increased compliance, and decreased adverse events.
Hartzler et al (2015) [23]	Tool Development	Step 1: 6, Step 2: 40	No quantifiable data.	The strategy for tool development was the engagement of healthcare providers to design a user-friendly patient care dashboard.
Badgeley et al (2016) [18]	Tool development	N/A	No quantifiable data.	No data provided; discusses tool development.
Ellsworth et al (2014) [19]	Tool development	23	No quantifiable data.	Large amount of clinical data needed to make clinical decisions; need options for viewing data based on clinical role.
Sebastian et al (2012) [24]	Tool development	N/A	No quantifiable data.	Tool development informed by qualitative data on satisfaction from interviews with neurosurgeons.
Swartz et al (2014) [22]	Tool development	N/A	No quantifiable data.	Survey and structured interview used to create tool. Tool has not been implemented. Better understanding of clinician needs can inform tool development.

^a^AWARE: Ambient Warning and Response Evaluation.

^b^NASA-TLX: NASA Task Load Index.

^c^MIVA: Medical Information Visualization Assistant.

^d^CLABI: central line associated blood stream infection.

^e^N/A: not applicable.

^f^PICU: pediatric intensive care unit.

Medical Information Visualization Assistant, v.2 (MIVA 2.0) is an EHR dashboard technology that uses a visualization engine to deliver multivariate biometric data by transforming it into temporal resolutions [13]. ICU clinicians can use selection menus to control the viewability of data in various time periods to assist with diagnosis and treatment [13]. The usability speed test identified no significant difference in time-on-task between the control group and the experimental group [13]. However, a significant difference was noted in speed with use of MIVA 2.0 [13].

### Clinician Satisfaction

Dziadzko et al (2016) studied the before-and-after implementation experience and satisfaction of ICU providers at two hospitals using the AWARE system [8]. Providers agreed that data gathering using the existing EHR system was difficult and time-intensive [8]. In a survey analysis, researchers found that prescribers were significantly more satisfied with the delivery of content and information output with AWARE due to the improvement of the presentation of information [8]. Bakos et al (2012) showed an increased use of the dashboard tool at Virginia Commonwealth University Health System throughout the first year of implementation, demonstrating clinician satisfaction with usage. Interviews further confirmed the benefit and helpfulness of using the tool as staff confirmed its usefulness in their workflow [14].

### Quality and Safety

Pageler et al (2013) discuss use of a checklist enhanced by the EHR and a unit-wide dashboard to improve compliance with an evidence-based, pediatric-specific catheter care bundle [15]. The researchers performed a cohort study with historical controls that included all patients with a central venous catheter at a 24-bed Pediatric ICU (PICU) in an academic children's hospital [15].

Central line associated bloodstream infection (CLABSI) rates decreased after the checklist intervention [15]. Analysis of specific bundle elements demonstrated decreased compliance with insertion bundle documentation. However, there was an increase in compliance with daily documentation of line necessity, dressing changes, cap changes, and port needle changes.

Shaw et al (2015) evaluated a real-time visual display that showed data on presence of consent for treatment, restraint orders, presence of urinary catheters, deep venous thrombosis (DVT) prophylaxis, Braden Q score, and medication reconciliation [16]. An automated EHR querying tool was created to assess compliance with a PICU safety bundle and querying of the EHR for compliance and updating of the dashboard automatically occurred every five minutes [16].

Baseline compliance and duration of noncompliance was established during three time periods: before activation of the dashboard, at one month following activation of the dashboard, and at three months after activation [16]. There was no difference between the three periods in presence of restraint orders, DVT prophylaxis, or development or worsening of pressure ulcers [16]. Between the first and third time periods, the median time from PICU admission to obtaining treatment consent decreased [16]. The number of patients with urinary catheters in place > 96 hours decreased significantly after the intervention [16]. The researchers concluded that a unit-wide dashboard could increase awareness for potential interventions, thereby affecting patient safety in a dynamic manner [16].

Although Bakos et al (2012) speculate that their visualization dashboard will contribute to having zero events of preventable harm to patients, employees and visitors; there is no quantifiable data to support this at this time [14]. Similarly, Hagland (2010) discusses the potential to improve patient safety, communication and clinician workflow using a new clinical dashboard without quantifiable results [17].

### Accuracy

Koch et al (2013) used the paper prototypes of their displays to measure situation awareness (accuracy of the participants’ answer). Nurses had a higher situation awareness and accuracy when using the integrated display versus the traditional display [11].

To evaluate the accuracy of, MIVA 2.0, Faiola et al (2015) used quantitative clinical decision-making task questions. The clinical decision-making accuracy test identified an overall significant improvement in accuracy of the eight-question test between the experimental versus control groups. Qualitative results were obtained from seven open-ended interview questions, wherein participants acknowledged the potential impact of MIVA 2.0 for reducing cognitive load and enabling more accurate decision-making [13]. Overall, a significant difference was noted in accuracy with use of MIVA 2.0 [13].

Dziadzko et al (2016) surveyed healthcare providers who reported an improvement in the accuracy of decision-making using AWARE, but no quantifiable data is available [8]. Using the .NET based application, Ahmed et al (2011) found that the median number of errors per provider decreased significantly for the novel user interface compared to the standard electronic medical record interface [12].

Farri et al (2012) evaluated the accuracy of using their spiral model software. The resulting differences in unretrieved patient information and accurate inferences were not statistically significant but suggested some improvement with the new information visualization tool [12]. Other observed effects of the tool included more intuitive navigation between patient details and increased effort towards methodical synthesis of clinical documents [12].

Scripps Clinic and Green Hospital demonstrated an improved accuracy of the healthcare provider “Heart Team” in clinical decision-making using 15 mock patients. However, a complete data analysis was not performed [20].

### Tool Development

Five articles did not focus on efficiency, quality and safety, accuracy and satisfaction outcomes but discussed their process of tool development. Their findings during visualization tool development are included in the discussion section below [18,19,22-24].

## Discussion

### Overview

**Figure 4:**
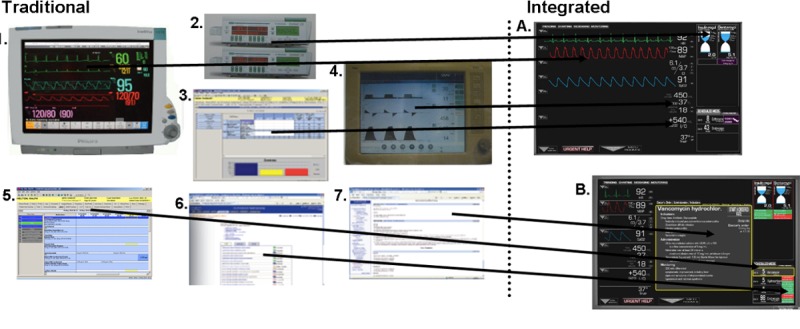
“(A) Nurses see an overview of the patient's vital signs, currently administered and scheduled medication, essential ventilation data, and fluid balance. (B) When selecting a medication they see medication compatibility with the other current and scheduled medication, and potential adverse effects.” [11].

**Figure 5:**
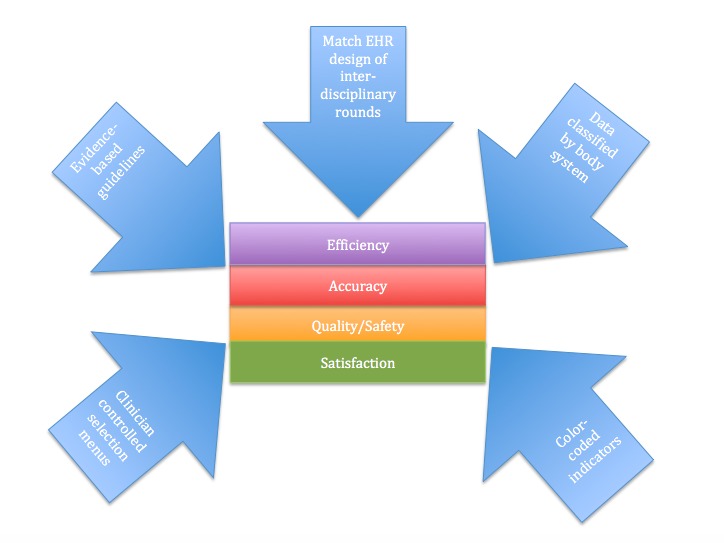
Summary of findings from the literature review. EHR: electronic health record.

The 17 articles included in this literature review demonstrate how efficiency, quality and safety, clinician satisfaction and accuracy can be improved using a visualization dashboard. These 17 articles share many themes regarding how each dashboard was designed and what user-friendly features are available when using the dashboard [8-24]. These themes are discussed below. With each idea outlined, a discussion of its application to prior visualization dashboard solutions and its implications for future studies follows. Application of these approaches, methods, and features may serve useful in future efforts related to this subject matter. A summary of findings from the articles is depicted in Figures 4 and 5.

### Human-Centered Design

A collaborative, human-centered approach informed the creation of several different dashboards. Structured survey and interview were used to inform iterative design and evaluate the final prototype of each dashboard design.

The IView dashboard was developed for use on three ICU's at the Children's Hospital at Pittsburgh and resulted from intensive clinician-IT team-based work and a collaborative relationship with the hospital's clinical IT vendor [13]. Qualitative measures regarding perceived patient safety, clinician workflow, and physician-nurse communications pointed to positive outcomes in all three categories [13].

Swartz et al (2014) discuss the creation of iNYP, a Java-based service-oriented web application, to meet the specific information needs of emergency medicine clinicians [23]. A combination of survey and structured interview were used to inform the development of this specialty-specific clinical dashboard [22].

Hartzler et al (2015) discuss the use of human-centered design methods to create visual displays of patient reported outcomes [23]. Targeted, iterative design activities were used to inform development of a dashboard that visually displays patient-reported pain and disability outcomes following spine surgery [23]. The Multi-signal Visualization of Physiology (MVP) was developed at the Neuroscience ICU of the National Neuroscience Institute in Singapore to provide a more visual, straightforward, and intuitive diagnosis process [24]. The MVP makes use of a polygram that incorporates live readings of physiological signs and colors to highlight different patient statuses [24].

### Interdisciplinary Approach

Nine articles mentioned use of an interdisciplinary approach in developing, testing, and implementing their visualization solution. The benefit of an interdisciplinary approach is that the varied professional perspectives and skills that come with different disciplines are integrated into each step of the process [8,10,13,14,17,19,20,22,23]

### Use of an Interactive Prototype

Prototyping is a useful process as it allows developers to strategize product design and obtain feedback from end users without the expansive investment of resources required to make changes in the EHR format [11-13,18,20,21,23]. While there are viable electronic prototyping options available, paper-based prototyping can be a useful, cost-effective solution in the early stages of product design [25]. A mixed evaluation process of quantitative and qualitative measures can be used to direct feedback from end user interaction with the prototype and improve design on subsequent revisions [11-13,18,20,21,23].

### Using Open-Source Technology vs Adapting a Third-Party Vendor's Electronic Health Record System

A team with limited resources may not be able to invest financial, temporal, and staff resources into developing a suitable product [26]. Those teams with limited resources may have to wait for a solution to stem from others using open-source technology or for the third-party vendor to provide an option that will be suitable [18,27]. Use of an open-source technology can allow more freedom for the user to develop and share their tool with others than when adjusting an EHR developed by a third-party vendor [18]. Ultimately, each team at a specific organization will decide which route aligns better with their own resources and goals, but the distinct opportunities and risks inherent with each option are important to consider.

Adapting a vendor's EHR system will require continual consultation with the vendor and there may be significant limitations imposed by the contract between the organization and the vendor [27].

### Application of Evidence-Based, Clinical Practice Guidelines to the Electronic Health Record

Clinical practice guidelines (CPGs) are intended to improve the quality, consistency, and effectiveness of care by applying evidence-based medicine [28]. A review of physician adherence to clinical practice guidelines suggested that as many as 38% of physicians consider clinical practice guidelines as inconvenient or too difficult to use [28]. Incorporation of clinical practice guidelines into the structure and display of the EHR may help improve convenience of access to practice guidelines and increase use in clinical decision-making [14-16].

### Using Open-Source Technology vs Adapting a Third-Party Vendor's Electronic Health Record System

A team with limited resources may not be able to invest financial, temporal, and staff resources into developing a suitable product [26]. Those teams with limited resources may have to wait for a solution to stem from others using open-source technology or for the third-party vendor to provide an option that will be suitable [18,27]. Use of an open-source technology can allow more freedom for the user to develop and share their tool with others than when adjusting an EHR developed by a third-party vendor [18]. Ultimately, each team at a specific organization will decide which route aligns better with their own resources and goals, but the distinct opportunities and risks inherent with each option are important to consider.

Adapting a vendor's EHR system will require continual consultation with the vendor and there may be significant limitations imposed by the contract between the organization and the vendor [27].

### Application of Evidence-Based, Clinical Practice Guidelines to the Electronic Health Record

Clinical practice guidelines (CPGs) are intended to improve the quality, consistency, and effectiveness of care by applying evidence-based medicine [28]. A review of physician adherence to clinical practice guidelines suggested that as many as 38% of physicians consider clinical practice guidelines as inconvenient or too difficult to use [28]. Incorporation of clinical practice guidelines into the structure and display of the EHR may help improve convenience of access to practice guidelines and increase use in clinical decision-making [14-16].

### Clinician Controlled Selection Menus

Allowing the clinician to adjust the data displayed in alignment with the preference and needs of that individual may further improve clinician satisfaction with the system [13,19]. This capability can also help meet the goal of reducing cognitive overload [13,19]. If clinicians can filter out information that is not pertinent to them, the remaining information will have improved visibility without obstruction from extraneous information [13,19]. The capability to filter information by location, service lines, and specific diagnoses may also serve useful to improve efficiency, accuracy and user satisfaction of clinicians managing many patients [13].

### Improved Display of Trends in Physiological Signs

In a setting such as an intensive care unit, the stability of a patient's condition can quickly deteriorate [2]. While clinicians have primary responsibility to assess their patient's condition and intervene appropriately, adding features to the EHR that can assist with this process can expedite these steps; improving efficiency [24]. With the vast array of physiological parameters under continuous monitoring in the ICU setting, improved display of data trends may improve the clinician's responsiveness in adding or weaning interventions based on the patient's changing condition [24].

### Classification of Data by Body System

Classification of data using a body system approach was a common decision for the EHR designs in this literature review [8,10]. By using a body system approach, clinicians can follow a systematic approach to optimizing the patient's holistic health. The design choice of matching the body system approach used by intensive care unit clinicians allows for congruency between the EHR display and cognitive organization of clinical information [8,10].

### Applicability of a Visualization Dashboard to Non—Intensive Care Unit Clinical Settings

While this literature review focused primarily on the application of a visualization dashboard to the intensive care unit setting, the same intervention could have benefit in other clinical settings as well [12,14,18,20-23]. The emergency department could be well suited for this intervention as the EHR could then assist in alerting clinicians to new results and a change in the patient's clinical status that modifies the plan of care [22]. Step-down units and inpatient floors may not have the same extent of clinical data as the intensive care unit setting but clinicians may still find benefit from features related to improved display of clinical information. Once a visualization dashboard is successfully implemented in the ICU setting, the dashboard can be modified, tested, and implemented in non-ICU clinical settings; working towards similar goals [18].

### Strengths and Limitations of Solutions

This literature review includes information on several visualization dashboards that have been tested with positive results from quantitative and qualitative analysis. These positive results support the potential benefits of a visualization dashboard solution to clinical practice environments. Limitations were noted in the following areas:

- The interpretation of what a visualization dashboard solution entails varied widely among the researchers of the different studies included.
- Many of the visualization dashboard solutions were evaluated with a solely qualitative approach, rather than with a quantitative or mixed methods approach.

- Some articles included details about the design, implementation, and evaluation processes, but did not include full detail on the data obtained.
- Some studies used a simulated setting in lieu of a live clinical setting, which means that results may differ when the solution is applied to a live clinical setting.
- Most studies tested a single solution in a single implementation setting, which limits the generalizability of the findings to other solutions and other implementation settings.

### Future Direction

Researchers can build on the findings, strengths, and limitations of the work identified in this literature review to bolster development, testing, and implementation of a novel visualization dashboard solution. Due to the relatively few studies conducted in this area, there is plenty of room for researchers to test their solutions and add significant information to the field of knowledge on this subject. An effective solution in this area can drive process improvement and improved patient outcomes for not only the initial setting of implementation, but also to any further clinical units and organizations that adopt the intervention.

### Conclusions

Overall, successful visualization dashboards utilized an interdisciplinary approach to develop a human-centered design. Dashboards were flexible and could be adjusted to the users’ preferences as well as organized based on body system, color-coded and adapted for clinician team rounding. These features are important due to the variety in patient population and the diverse way that clinicians interpret information. Utilizing these common themes to develop visualization tools for patient care has shown to improve efficiency, quality or safety, clinician satisfaction and accuracy in a variety of patient settings.

This section synthesizes the major findings of the 17 articles [8-24]. As discussed, visualization tools have the potential to impact accuracy, efficiency, user satisfaction, quality or safety of care in the ICU and other settings. Numerous factors such as clinician-controlled displays, organization by body system, an interdisciplinary design team and using open-source technology can result in successful implementation of a visualization dashboard. The findings, strengths, and limitations discussed in this section can drive future research efforts on visualization dashboard solutions.

### Design Recommendation based on Clinician Needs

Information needs varied based on patient population and clinical role. Key findings regarding clinician needs for the solution included: the application of evidence-based, clinical practice guidelines; clinician-controlled selection menus; the use of color-coded visual indicators; classification of data by body system and matching of EHR design to the process of interdisciplinary rounds. As demonstrated in the results section of this paper, the combination of the above components can allow for user-friendly dashboard designs that have the potential to impact accuracy, efficiency, user satisfaction and quality and safety of care.

## Abbreviations

AWARE: Ambient Warning and Response Evaluation
CLABI: central line associated blood stream infection.
CPGs: Clinical Practice Guidelines
DVT: Deep Vein Thrombosis
EHR: Electronic Health Record
ICU: Intensive Care Unit
MIVA: Medical Information Visualization Assistant.
MVP: Multi-signal Visualization of Physiology
NASA-TLX: NASA Task Load Index.
PICU: pediatric intensive care unit.
